# Membrane potential (V_mem_) measurements during mesenchymal stem cell (MSC) proliferation and osteogenic differentiation

**DOI:** 10.7717/peerj.6341

**Published:** 2019-02-08

**Authors:** Mit Balvantray Bhavsar, Gloria Cato, Alexander Hauschild, Liudmila Leppik, Karla Mychellyne Costa Oliveira, Maria José Eischen-Loges, John Howard Barker

**Affiliations:** Frankfurt Initiative for Regenerative Medicine, Johann Wolfgang Goethe Universität Frankfurt am Main, Frankfurt am Main, Hessen, Germany

**Keywords:** Membrane potential, Electrical stimulation, Mesenchymal stem cells, Osteogenic differentiation, Cell proliferation, V_mem_

## Abstract

**Background:**

Electrochemical signals play an important role in cell communication and behavior. Electrically charged ions transported across cell membranes maintain an electrochemical imbalance that gives rise to bioelectric signaling, called membrane potential or V_mem_. V_mem_ plays a key role in numerous inter- and intracellular functions that regulate cell behaviors like proliferation, differentiation and migration, all playing a critical role in embryonic development, healing, and regeneration.

**Methods:**

With the goal of analyzing the changes in V_mem_ during cell proliferation and differentiation, here we used direct current electrical stimulation (EStim) to promote cell proliferation and differentiation and simultaneously tracked the corresponding changes in V_mem_ in adipose derived mesenchymal stem cells (AT-MSC).

**Results:**

We found that EStim caused increased AT-MSC proliferation that corresponded to V_mem_ depolarization and increased osteogenic differentiation that corresponded to V_mem_ hyperpolarization. Taken together, this shows that V_mem_ changes associated with EStim induced cell proliferation and differentiation can be accurately tracked during these important cell functions. Using this tool to monitor V_mem_ changes associated with these important cell behaviors we hope to learn more about how these electrochemical cues regulate cell function with the ultimate goal of developing new EStim based treatments capable of controlling healing and regeneration.

## Introduction

Understanding, harnessing and controlling the body’s regenerative capabilities has long been among the most sought-after goals in medical research. Regenerative medicine, by fulfilling its promise of restoring full form and function to tissues and organs, could, for the first time, make it possible to *cure disease* rather than just treat the symptoms. Stem cells play a, if not the central role in regeneration as well as embryonic development (Daley, 2015; Mahla, 2016). The signals that regulate these cells are biochemical and/or bioelectric, the latter originating from the passage of positively and negatively charged ions across cell membranes. This active transport of charged ions in and out of cells gives rise to transmembrane voltage gradients or V_mem_. The V_mem_ across the membrane of cells that are in high proliferative states (embryonic, adult stem cells, cancer cells, etc.) have been shown to trend toward being more positive and are depolarized, while the V_mem_ of cells that are in low proliferative states (neurons, fibroblasts, skeletal muscle cells, fat cells, etc.) are more negative or hyperpolarized (Stillwell, Cone & Cone, 1973; Sundelacruz, Levin & Kaplan, 2008, 2009; Levin, 2012). During development, these V_mem_ changes across the membrane of embryonic stem cells constitute finely coordinated bioelectric signals that orchestrate embryonic growth throughout development. The presence and importance of this bioelectric activity on the surface of developing embryos, while poorly understood, has been clearly demonstrated on the surface of developing chick embryos and frog larva. Shi & Borgens (1995) measured distinct circular patterns of bioelectric flow around the spinal cords of developing chick embryos. When this electric flow was short circuited by implanting a copper wire adjacent to the electric fields, the chick developed without lower extremities, highlighting the importance of these bioelectric fields in development (Shi & Borgens, 1995). In a developing frog larva, Pai et al. (2015) chemically disrupted spatial gradients of the transmembrane potential (V_mem_) and induced forced hyperpolarization by mis-expression of specific ion channels which diminished the expression of early brain markers, causing absent or malformed regions in the embryo’s brain and death. In another study, Lan et al. (2014) depolarized V_mem_ of cardiac myocytes by adding potassium gluconate or Ouabain to the culture medium and found that depolarization of cardiac myocytes maintains cell proliferation. Also, Tseng & Levin (2013) demonstrated that body-wide pharmacological modulation of V_mem_ can induce functional regeneration of the froglet leg at a non-regenerative stage. Vandenberg, Morrie & Adams (2011) showed how membrane voltage and pH regionalization are required for craniofacial morphogenesis. Finally, studying bioelectricity in regeneration Borgens, Vanable & Jaffe (1977) were able to measure endogenous bioelectric current emanating from the stumps of amputated, regenerating newt limbs. They found that the intensity of these currents peeked at 4 days post amputation and then gradually subsided over the course of a week. In recent experiments in a rat limb amputation, and separately in a rat femur defect model we demonstrated that physiological levels of externally applied EStim, delivered to limb stumps and bone defects, respectively, significantly increased bone and cartilage regeneration and new vessel formation (Leppik et al., 2015, 2018).

Externally applied EStim has been used clinically to promote bone healing for many years. Only recently have we begun to understand the mechanisms, at a cellular level, by which EStim affects bone healing in this way. In several recent experiments others and we exposed cells, in culture, to externally applied EStim and observed major changes in cell behaviors like, proliferation (Guo et al., 2012; Sebastian et al., 2015; Qi et al., 2018), differentiation (Hernández et al., 2016; Mobini et al., 2017a; Eischen-Loges et al., 2018), migration (Jahanshahi et al., 2013; Yuan et al., 2014; Tai, Tai & Zhao, 2018) and over-all cell cycle progression (Griffin et al., 2013). While changes in endogenous bioelectric activity have been shown to play a crucial role in embryologic development and regeneration, and externally applied EStim has been shown to affect important cell functions involved in regeneration, the role V_mem_ plays in regulating these functions is still poorly understood. To better understand the role of V_mem_ in regeneration-related cell behaviors, in the present study, we used EStim to promote cell proliferation and osteogenic differentiation in adipose-tissue-derived mesenchymal stem cells (AT-MSC) and simultaneously tracked V_mem_ changes over time.

## Materials and Methods

*Groups:* Rat AT-MSC were allocated into “cell proliferation” and “osteogenic differentiation” groups. Cells in the proliferation group were cultured in normal medium and were further divided into two groups: Cells that received no EStim (controls) and cells exposed to EStim. Cells in the osteogenic differentiation group were cultured in osteogenic medium and were further divided into two groups: Cells that received no EStim (controls) and cells exposed to EStim. All cells were stained with the membrane potential (V_mem_) sensitive florescent dye, DiBAC_4_(3), and imaged at 0, 7, 14, and 21 days, and cell proliferation and osteogenic differentiation were measured at the same time points.

*Cell preparation and culture:* Rat AT-MSC were purchased from Cyagen Biosciences (Cat. No. RASMD-01001) and stored in liquid nitrogen at −196 °C, then on the day of the experiment they were thawed, cultured, and expanded to reach the desired number, according to the cell provider’s instructions. To achieve the appropriate number, cells were cultured until they reached 80% confluency and then expanded over 6–8 passages. Cells from passage 6–8 were seeded in 6-well cell culture plates (TPP, Trasadingen, Switzerland) at a density of 90,000 cell/cm^2^ in cell growth normal medium consisting of Dulbecco’s Modified Eagle Medium, GlutaMAX 1 g/L D-Glucose, 10% Fetal Calf Serum, and 1% Penicillin/Streptomycin (10 U/ml), all obtained from GibcoR (Gaithersburg, MD, USA). The seeded 6-well plates were then placed in a humidified incubator at 37 °C with 5% CO_2_ and 5% O_2_. These cells were used for cell proliferation experiments. For osteogenic differentiation experiments, the cell growth medium in which they had been cultured until this point was replaced with osteogenic cell growth medium, complemented with 10^−7^ M dexamethasone, 10 mM glycerophosphate, and 0.05 mM ascorbic acid-2-phosphate, all obtained from Sigma-Aldrich (Heidelberg, Germany). The culture medium was replaced two times per week.

**Figure 1:**
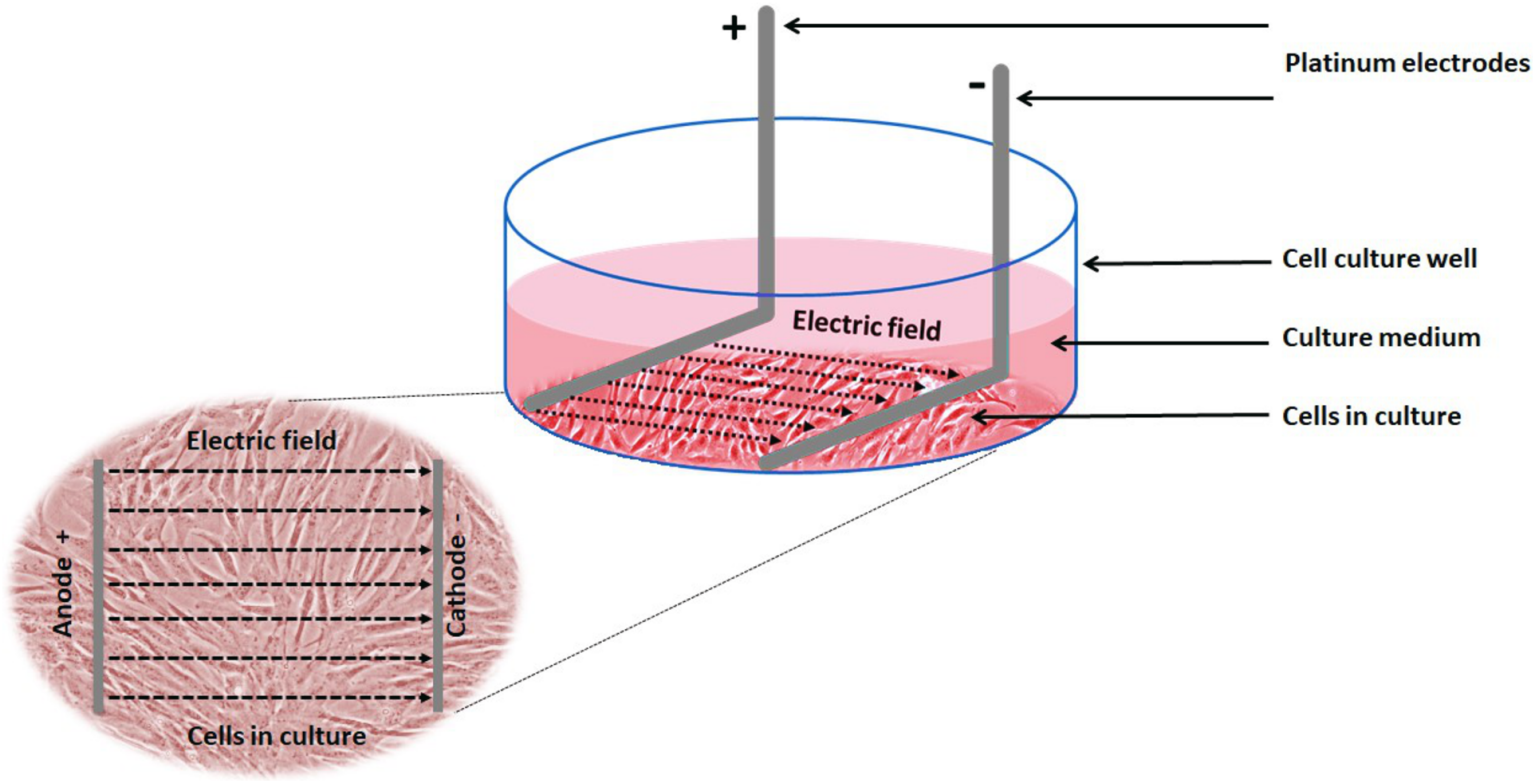
Schematic showing the experimental setup and electric field relative to the cells. Experimental setup showing 1 of 6 wells in our EStim cell culture chamber. AT-MSC seeded in culture medium in a well. L-shaped platinum electrodes, in contact with the bottom of the well, completely immersed in culture medium. Inset image showing cultured cells between anode (+) and cathode (−) in electric field. The image has been edited from (Mobini et al., 2017a, 2017b).

*Electrical stimulation of cells:* Electrical stimulation (EStim) was applied by means of a purpose-built DC EStim cell culture chamber described elsewhere (Mobini, Leppik & Barker, 2016). Briefly, the EStim chamber consists of a standard 6-well plate with a lid equipped with six pairs of platinum and silver electrodes connected to an electrical power supply (e.g., Triple Output Programmable DC Power Supplier (Supply-Model 9130; B&K Precision, Yorba Linda, CA, USA) (Fig. 1). Using this setup cells were exposed to 100 mV/mm of DC EStim for 1 h/day over the entire duration of the 21-day experiment.

*V_mem_ measurements:* To visualize and measure V_mem_ changes at the predetermined measurement time points (0, 7, 14, and 21 days) during cell proliferation and osteogenic differentiation, cells were dyed with the anionic voltage-sensitive dye, Bis-(1,3-diethylthiobarbituricacid) trimethine Oxonol (DiBAC_4_(3), Invitrogen, Carlsbad, CA, USA), whose uptake by cells is voltage dependent. Higher dye uptake is seen in more depolarized cells (Sundelacruz, Levin & Kaplan, 2008; Dibac, Adams & Levin, 2014). V_mem_ changes were visualized and measured using fluorescence microscopy, as described by Adams & Levin (2014). For each measurement a fresh solution of 10 mM DiBAC_4_(3), in DMSO was prepared and diluted to 0.5 mM in Hank’s Buffered Salt Solution (HBSS, Invitrogen, Carlsbad, CA, USA). After adding the dye, the cells were left for 30 min in an incubator at 37 °C, then washed two times using PBS at room temperature and imaged using a Nikon Eclipse Ti-E Inverted Microscope. The DiBAC_4_(3) dye was excited with a 420 nm light and the fluorescence images were captured at 520 nm by a non-descanned photomultiplier tube, controlled by NIS Element Software. The captured images were saved as a bright field (BF) image and for every BF image, a flatfield image (FF) (made by defocusing the image) and a dark field (DF) image (made by closing the shutter) were taken. These three images were later used for corrections (see below). All samples were imaged on the same day to minimize time dependent variations. Since fluorescence intensity was quantified for each image, the gain, exposure time, and offset settings of the microscope were kept constant over the duration of each experiment.

*Image correction:* Image correction was done as previously described (Adams & Levin, 2012) using the arithmetic function of NIS Elements imaging software. To correct the images, the corresponding pixels in the three images (BF, FF, DF) were subtracted and a new image of the difference was generated. Each image correction was done as follows:

- Raw BF image – DF image = DF corrected BF image
- FF image – DF image = DF corrected FF image
- DF corrected BF image – DF corrected FF image = Corrected final Image

*Image quantification:* Image quantification was done using NIH’s publicly available software, ImageJ. The level of fluorescence of the corrected images was quantified by selecting highly florescent areas within the cell as described in McCloy et al. (2014). Next to these areas, outside the cell an area with no fluorescence was selected to serve as background. Finally, to calculate corrected total fluorescence the following formula was used:

$$\eqalign{& {\rm{Corrected\, total\, cell\, fluorescence\,(CTCF)}} \cr &{\rm{ = }}\,{\rm{Integrated\,density}} - ({\rm{Area}}\,{\rm{of}}\,{\rm{selected}}\,{\rm{cell}}*{\rm{Mean}}\,{\rm{fluorescence}}\,{\rm{of}}\,{\rm{background}}\,{\rm{readings)}} \cr} $$

*Cell proliferation:* To measure cell proliferation cell number was counted indirectly by estimating dsDNA content according to the Quant-iT TM PicoGreen^®^ dsDNA Reagent and Kits protocol (29851; Molecular Probes, Inc., Eugene, OR, USA) (Pabbruwe, Stewart & Chaudhuri, 2005). Briefly, cells were washed two times with PBS, treated with lysis buffer (400 mM potassium phosphate buffer, 2% Triton X100, 10 mM EDTA, pH 7.0), and cell lysates were used for DNA content measurements. A serial dilution of a known amount of AT-MSC was lysed with lysis buffer and used to create a calibration curve showing the correlation between cell number and fluorescence. This latter procedure allowed us to indirectly determine the number of cells in the cultured wells via the calibration curve and measurement with PicoGreen^®^.

*Osteogenic differentiation:* Osteogenic differentiation was measured using Alizarin Red that stains calcium deposits in the cells. Cultured cells were washed twice with PBS and fixed with 4% paraformaldehyde (Sigma-Aldrich, München, Germany) solution in PBS for 30 min. Alizarin Red S (Sigma-Aldrich, München, Germany) solution (2% in PBS) was added to the fixed cells, incubated at room temperature for 30 min, and rinsed with deionized water repeatedly. Images were captured with a light microscope (CKX53, CellSens Entry 1.9 Software; Olympus, Tokyo, Japan) at a magnification of 10×. Red color deposits in the images indicate the presence of the complex formed by calcium deposits and the alizarin red. ImageJ Software was used to quantify the intensity of the red color as described in Camci-Unal et al. (2016).

*Data analysis and statistics:* All experiments were performed in triplicate and Microsoft Excel (2016) was used for all statistical assessments. In most cases, data is presented as the mean value ± standard deviation unless otherwise indicated. The statistical significance of differences between groups was analyzed by one-way ANOVA and student *t*-test using Microsoft Excel 2016. Significance level was set at *p* < 0.05.

## Results

### Cell proliferation and V_mem_ measurements

**Figure 2:**
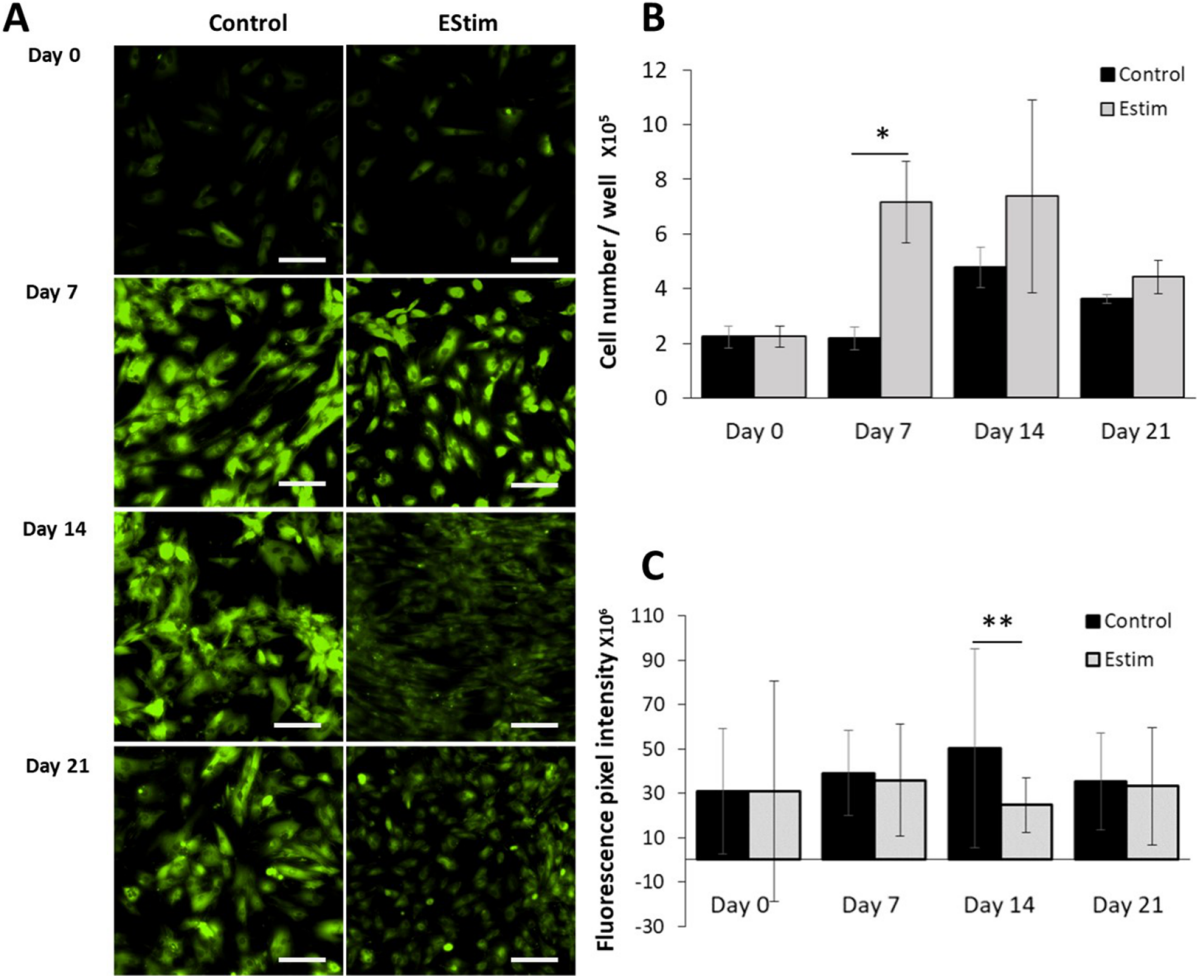
AT-MSC proliferation and V_mem_ measurements. (A) Representative fluorescence images of V_mem_ in EStim-treated and non-treated AT-MSC at days 0, 7, 14, and 21. (B) Graph of proliferation (cell number) of EStim-treated and non-treated AT-MSC at days 0, 7, 14, and 21. (C) Graph of fluorescence intensity (V_mem_), of EStim-treated and non-treated AT-MSC at days 0, 7, 14, and 21. V_mem_ (fluorescence intensity) was significantly (*p* < 0.01) higher in EStim vs. non-treated AT-MSC at day 14. Bar graphs represent mean pixel intensity with standard deviation (*n* = 45, 5–10 cells/Image from 15 images). Asterisks indicate degree of significant differences between groups at the same time points. *p < 0.05, **p < 0.01.

The cells, used for cell proliferation experiments, were cultured in cell growth normal medium. Cell proliferation and V_mem_ in AT-MSC exposed to, and not (controls) exposed to EStim were measured using PicoGreen^®^ assay and DiBAC_4_(3) voltage-sensitive fluorescent dye, respectively (Fig. 2). Proliferation was significantly (*p* < 0.05) increased by 3.5-fold in EStim-treated samples on day 7. It also increased by two-fold, though not significantly, on day 14, and there was no difference between EStim-treated and control cells on day 21 (Fig. 2B). V_mem_ values (fluorescence) were the same as controls on days 7 and 21 and were significantly (*p* < 0.01) decreased in EStim treated cells on day 14 (Figs. 2A right and 2C), and (Figs. 2A left and 2C).

### Osteogenic differentiation and V_mem_ measurements

**Figure 3:**
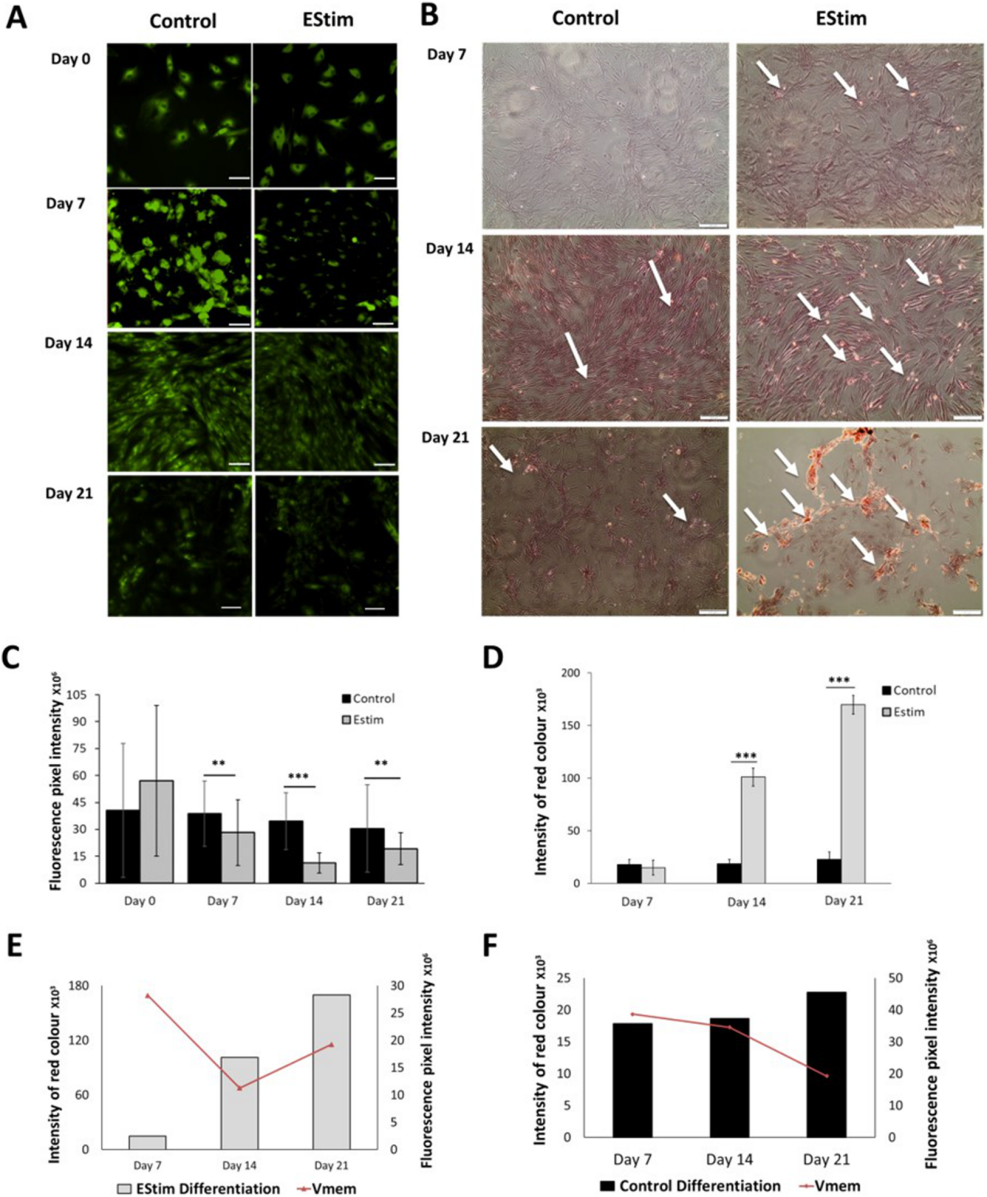
AT-MSC osteogenic differentiation and V_mem_ measurements. (A) Representative fluorescence images of EStim-treated and non-treated AT-MSC during osteogenic differentiation at days 0, 7, 14, and 21. (B) Representative images of Alizarin Red stained calcium deposits in EStim-treated and non-treated AT-MSC during osteogenic differentiation at days 7, 14, and 21. (C) Graph of fluorescence intensity (V_mem_) values of EStim induced osteogenic differentiation in AT-MSC at days 0, 7, 14, and 21. (D) Graph of intensity of red coloration (calcium deposits) in EStim-treated and non-treated AT-MSC at days 7, 14, and 21. (E) Graph of changes in intensity of red coloration (calcium deposits) (black bars) with superimposed fluorescence intensity (V_mem_) (red line) in non-treated AT-MSC at days 7, 14, and 21. (F) Graph of changes in intensity of red coloration (calcium deposits) (grey bars) with superimposed fluorescence intensity (V_mem_) (red line) in EStim-treated AT-MSC at days 7, 14, and 21. Bar graphs represent mean pixel intensity with standard deviation (*n* = 45, 5–10 cells/Image from 15 images). Asterisks indicate degree of significant differences between groups at the same time points (*p < 0.05, **p < 0.01, ***p < 0.001).

The cells, used for osteogenic differentiation experiments, were cultured in cell growth osteogenic medium. Osteogenic differentiation and changes in V_mem_ in EStim treated and non-treated (control) AT-MSC were measured using Alizarin Red and the voltage-sensitive fluorescent dye DiBAC_4_(3) respectively, the results of which are shown in Fig. 3. We found that fluorescence profiles (V_mem_ values) significantly decreased on for day 7 (*p* < 0.01), day 14 (*p* < 0.001) and day 21 (*p* < 0.01) (hyperpolarized) during osteogenic differentiation (Fig. 3A). Accordingly, the EStim treated cells, as the osteogenic differentiation progressed, had lower (less bright) fluorescence profiles than the controls (Figs. 3A, 3C and 3E). The fluorescence profile (V_mem_) of cells not exposed to EStim (controls) was high at all measurement time points decreasing only on day 21 (Figs. 3A, 3C and 3F). Cells treated with EStim showed an increase in calcium deposits (osteogenic differentiation) already beginning on day 7 and increasing until the end of the experiment, on day 21 (Fig. 3B, right). In cells not receiving EStim (controls), there were little or no calcium deposits at the same measurement time points. We quantified the red color of calcium deposition using ImageJ Software and found that osteogenic differentiation (calcium deposits) was significantly increased at day 14 (*p* < 0.001) and day 21 (*p* < 0.001) in EStim-treated samples (Fig. 3D), and there was no difference between EStim-treated and control cells on day 7 (Fig. 3D).

## Discussion

Voltage gradients across the plasma membrane or V_mem_ have been shown to be associated with cell behaviors like cell proliferation (Blackiston, McLaughlin & Levin, 2009; Sundelacruz, Levin & Kaplan, 2009), differentiation (Sundelacruz, Levin & Kaplan, 2008), migration (McCaig et al., 2005; Özkucur et al., 2011; Yang & Brackenbury, 2013) and over-all cell cycle progression, all behaviors that play a crucial role in tissue regeneration (Sundelacruz, Levin & Kaplan, 2009). During periods of differentiation, cell V_mem_ has been observed to be hyperpolarized, maintaining values that fluctuate around −90 mV (Levin, 2011). As was first shown by Levin et al., cellular differentiation causes hyperpolarized cellular states and as the process of differentiation continues, the cellular population as a whole becomes more hyperpolarized (Sundelacruz, Levin & Kaplan, 2008, 2009).

In recent in vitro studies others (Gittens et al., 2013; Hu et al., 2014; Kwon, Lee & Chun, 2016; Zhang, Neoh & Kang, 2017) and we (Mobini, Leppik & Barker, 2016; Mobini et al., 2017a; Eischen-Loges et al., 2018) showed that these same cell behaviors can be induced by exposing cells to externally applied DC EStim. Zhang, Neoh & Kang (2017) exposed human AT-MSC to 200 μA of DC EStim for 4 h/day for 7, 14, and 21 days and observed enhanced osteogenic differentiation and increased migration of cells into scaffold material. Similarly, in previous in vitro studies we found that 100 mV/mm of DC EStim for 1 h/day for 7 days stimulated cell proliferation and osteogenic differentiation in bone marrow derived MSC (Mobini, Leppik & Barker, 2016; Mobini et al., 2017a). In the present study we used EStim to stimulate cell proliferation and osteogenic differentiation in AT-MSC and at the same time tracked V_mem_ changes.

One of the hallmarks of regeneration is cell proliferation. Cell proliferation is a multi-step event regulated by a system of checkpoints at different phases of the cell cycle (Romar, Kupper & Divito, 2016). It is known that most non-proliferative cells (e.g., Nerve cells) have hyperpolarized (more negative) V_mem_, while most proliferative cells (e.g., Cancer cells) have depolarized (less negative) V_mem_ values (Lan et al., 2014). Cells are physically transferred to in vitro culture from an in vivo environment using cell extraction techniques, they tend to undergo spontaneous proliferation, which is accompanied by V_mem_ depolarization (Sundelacruz, Levin & Kaplan, 2009). In our experiments EStim treated cells showed higher levels of proliferation than non-EStim treated controls at all the measured time points (Fig. 2B). During this period, we tracked V_mem_ of the proliferating AT-MSC and found that values of the EStim treated cells showed no significant decrease, except on day 14 (Fig. 2C). During the same period the non-EStim treated cells showed a similar non-changing pattern and were strongly depolarized (high fluorescence) from days 7 to 21. These observations coincide with those of others who showed that cell proliferation is mostly accompanied by V_mem_ depolarization (Sundelacruz, Levin & Kaplan, 2009).

In the present study, as expected we found that in EStim treated cells osteogenic differentiation (calcium deposition by differentiated early osteoblasts) already began on day 7 and continued until day 21 (Fig. 3B). During the same period V_mem_ values were low (hyperpolarized). Taken together with our fluorescence data tracking V_mem_, we saw that a more negative V_mem_ correlates well with differentiation, as seen in the EStim group. This correlates with findings by others (Sundelacruz, Levin & Kaplan, 2009), in which lower V_mem_ values were shown to correlated with higher levels of differentiation. Our findings agree with those of Levin, et al. who found a clear relationship between hyperpolarized cellular states and high levels of cell differentiation. Taken together and compared to both the EStim and control groups, it is clear that AT-MSC hyperpolarization correlates with cell differentiation. Overall, our findings agree with previous work described in the literature (Sundelacruz, Levin & Kaplan, 2008). In said experiments by others cell proliferation and differentiation and the corresponding V_mem_ changes were induced using chemical interventions, that is, K^+^-ATP-channel openers pinacidil and diazoxide (Sundelacruz, Levin & Kaplan, 2008)_._ In contrast, in the present study we used DC EStim to stimulate proliferation and osteogenic differentiation, and also observed the corresponding changes in V_mem_. The fact that cell proliferation and differentiation and the corresponding V_mem_ changes can be stimulated both chemically (K^+^-ATP-channel openers pinacidil and diazoxide), and physically (DC EStim), in the present experiments, indicates that this correlation is robust. While based on this data we cannot say how EStim might affect V_mem_, in studies by Valič et al. (2003) and Taghian, Narmoneva & Kogan (2015), the authors speculate that DC EStim acts on the permeability of the ion channels through which electrically charged molecules pass the cell membrane.

## Conclusion

In summary, by monitoring V_mem_ signaling during changes in stem cell behaviors we hope to gain a better understanding of the mechanisms by which these electrochemical cues regulate stem cell function. In future experiments we will determine if the observed V_mem_ changes control proliferation and differentiation in the cells or if it is a consequence of these important cellular functions. This knowledge could lead to the development of new EStim-based therapies that optimize stem cell function in regenerative medicine-based treatments.

## Supplemental Information

10.7717/peerj.6341/supp-1 Supplemental Information 1 AT-MSC proliferation and V_mem_ measurements.

10.7717/peerj.6341/supp-2 Supplemental Information 2 AT-MSC osteogenic differentiation and V_mem_ measurements.

## Additional Information and Declarations

### Data Availability

The following information was supplied regarding data availability: The raw measurements are available in the Supplemental Files 1 and 2. Figure 2 shows raw data regarding AT-MSC proliferation and Fig. 3 regarding AT-MSC osteogenic differentiation.
